# Enhancing electrochemical discharge drilling performance in mild steel sheet using KMnO_4_ mixed electrolytes

**DOI:** 10.1038/s41598-025-23399-9

**Published:** 2025-11-12

**Authors:** Sekar Tamilperuvalathan, Vinoth Varadharaju, Vijay Manoharan, Sakthivel Rajamohan, Dhinesh Balasubramanian, Utku Kale, Artūras Kilikevičius, Vilma Locaitienė

**Affiliations:** 1 Department of Mechanical Engineering, Government College of Technology, Coimbatore, India; 2 Faculty of Mechanical Engineering, Dhirajlal Gandhi College of Technology, Salem, India; 3 Faculty of Mechanical Engineering, Dhanalakshmi Srinivasan Engineering College, Perambalur, India; 4 Department of Mechanical Engineering, Government College of Technology, Coimbatore, India; 5 Department of Mechanical Engineering, Mepco Schlenk Engineering College, Sivakasi, Tamil Nadu India; 6 Department of Port Engineering, Lithuanian Maritime Academy (LMA), Vilnius Gediminas Technical University, Klaipeda, Lithuania; 7 Department of Aeronautics and Naval Architecture, Faculty of Transportation Engineering and Vehicle Engineering, Budapest University of Technology and Economics, Műegyetem rkp. 3, Budapest, H-1111 Hungary; 8 Mechanical Science Institute, Vilnius Gediminas Technical University, Plytinės g. 25, Vilnius, LT-10105 Lithuania

**Keywords:** ECDD, KMnO_4_, NaOH, KOH, M.S sheet, MRR, TWR, HAZ, Chemistry, Energy science and technology, Engineering, Materials science

## Abstract

The electrochemical discharge drilling (ECDD) process is a hybrid technique combining electrochemical and electric discharge drilling, essential for machining advanced engineering materials like metal sheets, composites, and ceramics. This study introduces potassium permanganate (KMnO_4_) mixed with sodium hydroxide (NaOH) and potassium hydroxide (KOH) electrolytes to enhance the ECDD process. Key findings demonstrate that a 5% KMnO_4_ in 95% NaOH electrolyte significantly improves dimensional accuracy, achieving a hole diameter deviation of only 0.74% from the 1.75 mm tool electrode diameter. This mixture also minimizes stray corrosion and localized heating, enhancing precision. The highest material removal rate (MRR) of 3.33 mg/min was achieved with this combination, representing a 4.1% increase over plain NaOH. Additionally, the tool wear rate (TWR) was reduced by 70% to 5.5 mg/min. The reduced tool wear observed in this study might be attributed to probable tool surface modifications, including the formation of protective oxide layers and oxygenated functional groups. Such modifications can enhance heat dissipation and stabilize the tool–electrolyte interface, thereby contributing to improved tool performance. The interaction between KMnO_4_ and NaOH improved electrolyte stability, resulting in lower Heat Affected Zones (HAZs).

## Introduction

Electrochemical discharge drilling (ECDD) integrates the efficiency of electrical discharge drilling (EDD) with the precision of electrochemical drilling (ECD). However, because multiple energy sources are simultaneously involved in material removal, accurately and promptly detecting breakthrough remains a significant challenge^1^ The ECDD, as a hybrid process combining electric discharge drilling and ECD, addresses several limitations, such as material conductivity constraints, low material removal rate (MRR), high tool wear rate (TWR), heat-affected zones (HAZ), and poor dimensional and geometrical accuracy. The process relies on applied voltage, duty cycle, electrolyte concentration, temperature, chemical characteristics, current density, passivation layer characteristics, and material properties of electrodes and auxiliary anodes to achieve optimal MRR, TWR, and accuracy with minimal HAZ^2^. This process is extensively employed in making of high-aspect-ratio micro-holes, which are crucial for Micro-electro-mechanical systems (MEMS) and miniaturized electronics. The versatility and scalability of ECDD for MEMS device fabrication using lithography-free patterning and multi-hole formation in insulating substrates^3^. The selected electrolytes for ECDD are divided into two categories namely neutral solutions, which include sodium nitrate (NaNO₃) and sodium chloride (NaCl), and alkaline solutions, comprising sodium hydroxide (NaOH) and potassium hydroxide (KOH), as well as a mixture of both NaOH and KOH. Experimental results indicate that, compared to the traditional Newtonian fluid KOH electrolyte, a non-Newtonian fluid electrolyte significantly reduces the impact force on the gas film, resulting in greater stability^4^.

Although NaOH is the most preferable electrolyte medium for ECDD owing to its excellent conductivity and alkalinity, it poses several challenges including hazardous handling, formation of passivating oxide layers, electrolyte instability during spark discharge, and significant tool wear and heat-affected zones. These limitations necessitate the identification of novel electrolyte additives that can sustainably enhance machining efficiency, repeatability, and dimensional accuracy while reducing environmental and safety concerns. In this context, potassium permanganate (KMnO₄), a strong oxidizing agent, has been introduced as an innovative additive to address these gaps. KMnO₄ actively breaks down passivating films on the workpiece surface, stabilizes the gas film during discharge by generating oxygen, and improves the overall electrochemical environment, resulting in higher MRR, reduced tool wear, and minimized heat-affected zones, as demonstrated in this study^5^.

The combined mechanism of electrochemical dissolution and electric discharge erosion facilitates the machining of materials with high strength and temperature resistance, which are difficult to machine using conventional methods^6^. Research has shown that machining voltage significantly impacts tool electrode wear, with pure water as the electrolyte improving the process for stainless steel^7^. Tool loss is in the form of localized melting or gasification at elevated temperature^8^. Mixed alkaline electrolytes have been reported to enhance machining performance by improving ionic conductivity and stabilizing discharge phenomena. For instance, studies on borosilicate glass demonstrated that mixed electrolytes improve MRR, surface quality, and dimensional accuracy relative to single electrolyte solutions. Incorporating oxidizing agents like KMnO₄ alongside NaOH and KOH electrolytes further mitigates challenges related to passivation and electrolyte stability in metallic machining environments. This approach enhances machining accuracy, MRR, and tool life^9^. Spring actuation mechanisms and high-speed electrodes enhance the process for difficult-to-cut materials by disrupting insulating films and increasing machining efficiency^10^. Tool electrode roughness also affects geometric characteristics, with smoother electrodes reducing overcut and surface damage^11^. High voltage, however, can significantly reduce tool life due to the generation of high thermal energy sparks^12^. In alumina micromachining, the use of multi-tip and arrayed electrodes significantly impacts machining uniformity and tool wear characteristics in ECDD. The effects of electrode geometry and process parameters such as pulse frequency on tool life and machining consistency has providing pathways to enhance electrode longevity and machining quality^13^. Blind hole drilling also presented challenges, with tool electrode wear reducing hole depth accuracy. Methods like using high-conductivity electrolytes, electro-deposition, nanosecond voltage pulses, and side-insulated electrodes can reduce wear and improve accuracy^14^. Wettability of tool electrodes, influenced by surface roughness, affects the gas film’s stability, which is crucial for machining performance^15^. Advanced hollow and tubular electrode designs facilitate improved electrolyte flow and debris evacuation, which enhance MRR during deep drilling of insulating materials. The implementation of notch-shaped tubular electrodes and controlled electrolyte injection has been shown to substantially increase machining efficiency in macro-scale hole fabrication^16^.

The chemical reaction between two electrodes can be expected depending on the selection of the electrolyte based on its nature of passivating and non-passivating characteristics^17^. Introducing nanoparticles like Cu and Al_2_O_3_ into electrolytes improved electrical and thermal conductivity, enhancing hole depth and tool life despite slightly increasing entrance overcut^18^. Lower electrolyte concentrations enhance chemical etching, which can cause surface wrinkling^19^. Research on the electrolytes used for Electrochemical Discharge Machining (ECDM) reported that two alkaline electrolytes namely NaOH and KOH were mixed at 15 and 25 wt% concentrations and observed more electrical conductivity than KOH and NaOH separately at the same concentrations^20^. Mixed electrolytes of NaOH and KOH have been shown to improve MRR, with ion enrichment playing a key role in the process^21,22^. Orbital motion of the tool electrode significantly enhances drilling performance^23^. The ultrasonic-assisted electrochemical discharge machining (UA-ECDM) has emerged as a promising advancement to enhance machining performance. The application of ultrasonic vibrations to the electrolyte significantly improves debris removal and agitation within the machining zone, thereby increasing MRR and enhancing surface quality^24^. The ECDM has been effectively used to machine challenging materials like alumina, showing that ultrasonic vibrations significantly increase hole depth^25^. Gravity-feed drilling in glass using ECDD is controlled by discharge and hydrodynamic regimes, affecting drilling speed^26^. Applied voltage and tool feed rate are critical parameters for achieving minimal overcut and taper in silicon wafer drilling^27^. Borosilicate glass machining studies using design of experiments and response surface modeling optimize MRR^28^. Despite advancements, further intensive research is needed to understand the mechanism of ECDD process completely. Factors like applied voltage, inter-electrode gap, temperature, electrolyte concentration, electrode shape and material, and power supply nature significantly influence ECDD^29^. Time-varying current observations help propose basic mechanisms of temperature rise and material removal^30,31^. There is still confusion regarding the distinction of the discharge current from the electrochemical reaction current in ECDM^32^. Adding abrasive particles to the electrolyte reduces slit expansion and improves surface roughness by increasing the critical voltage and reducing discharge energy^33^.

Adaptive tool feed systems, which withdraw the tool when it contacts the work material, improve geometric accuracies of micro-holes^34^. Gas film quality, influenced by electrolyte concentration, electrode distance, and machining time, is critical for controlling overcut in ECDD^35,36^. For non-conductive fibrous composites in aerospace and marine industries, ECDD addresses challenges of high tool wear and poor surface roughness^37^. A reversible motion-wire electrochemical spark machining (RM-WECSM) has been developed for curve profile cutting, especially on non-conducting workpieces such as Al_2_O_3_-Epoxy nanocomposite of 3.5 mm thickness and analysed the effects of NaOH concentration, applied voltage, reversible wire velocity, pulse on-and-off time on MRR and average surface roughness^38^. Studies on soda-lime glass using ECDD show that voltage significantly influences machined depth and tool electrode effectiveness^39^. Efforts to improve ECDD performance include introducing KMnO_4_ with NaOH and KOH electrolytes. KMnO_4_, as a strong oxidizing agent, prevents passivating layer formation, enhancing machining efficiency by breaking down the iron surface more effectively. This innovative approach addresses the predominant challenge of the gas insulation layer, advancing ECDD’s effectiveness.

## Methodology and experimentation

The formation of cast layers, stray corrosion, and gas insulation films across the gap between the tool electrode and workpiece significantly influence the geometric and dimensional accuracies of drilled holes in the ECDD process. Despite the incorporation of numerous mechanisms to improve performance, this research specifically introduces KMnO_4_ mixed with NaOH and KOH electrolytes at various molarities.

The selection of 5% KMnO₄ concentration was determined based on a series of preliminary experiments aimed at optimizing the balance between oxidative action, drilling performance, and electrolyte stability. Initial trials were conducted by varying KMnO₄ content between 1% and 7% by weight in NaOH and KOH media. Results revealed that concentrations above 5% led to excessive oxidation, increased stray corrosion, and instability of the electrolyte. Conversely, concentrations below 5% did not produce notable improvements in MRR, TWR, or hole dimensional accuracy. The 5% KMnO₄ level consistently delivered the lowest tool wear and deviation from the target hole size, while maximizing MRR and minimizing HAZ. Thus, this value was selected as optimal for further investigation and full factorial comparison.

**Fig. 1:**
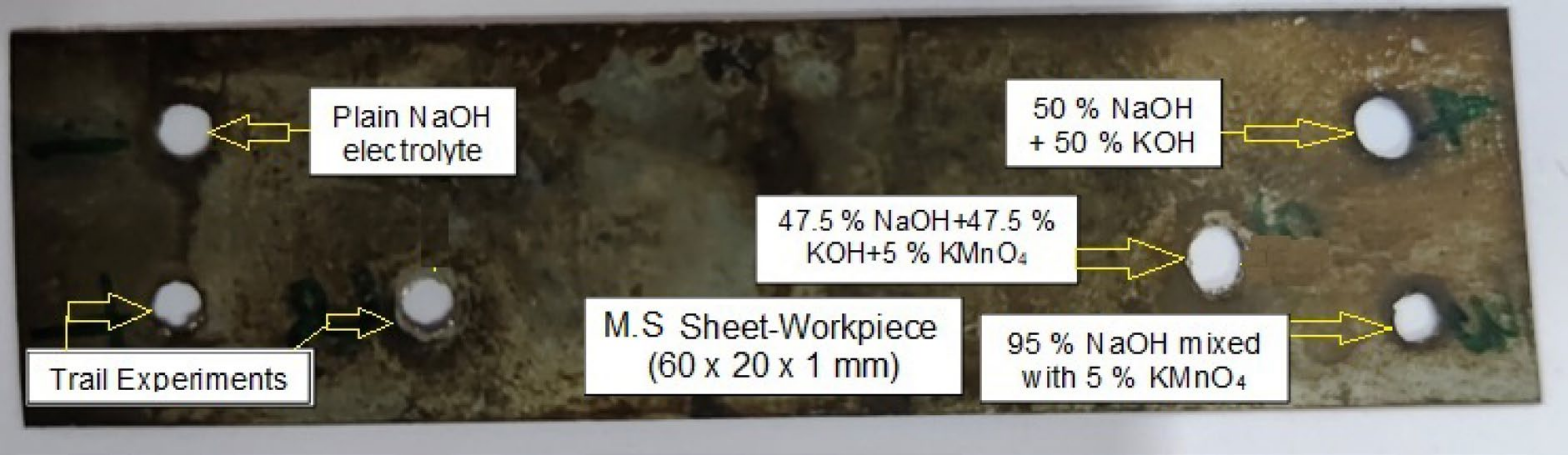
ECDD of MS sheet using various electrolyte combinations with/without KMnO_4_.

Other combinations, including 100% NaOH, 95% NaOH + 5% KMnO₄, 50% NaOH + 50% KOH, and 47.5% NaOH + 47.5% KOH + 5% KMnO₄, were selected to systematically examine the impact of pure, mixed, and oxidizer-enriched alkaline electrolytes. NaOH is a standard electrolyte for ECDD due to its high conductivity but presents issues of passivation and handling safety. The blending of NaOH and KOH was included based on literature evidence that mixed alkaline electrolytes offer increased ion mobility and process stability compared to single-component systems. Incorporation of KMnO₄, a strong oxidizer, in both single and mixed alkaline solutions allowed for evaluation of its effects under different ion environments. This systematic approach enabled identification of the optimal recipe that resulted in enhanced dimensional accuracy, reduced tool wear, and controlled thermal effects in the ECDD process. This approach aims to enhance the drilling of holes in a Mild Steel (M.S) sheet with dimensions of 60 × 20 × 1 mm, as shown in Fig. 1.

Zirconia, a widely preferred engineering ceramic for healthcare and modern engineering applications, was machined using ECDM with NaOH as the electrolyte. It was reported that using a voltage of 100 V and duty cycle of 50% resulted in a higher MRR and lower surface roughness^40^. This work is performed for material removal in drilled holes by ECDM processing. The effects of key process parameters—voltage, electrolyte concentration, and duty cycle—on the performance in ECDM of high-speed steel milling cutter have been studied across various levels^41^. Based on the literature review, previously published research, and trial-and-error methods, the key influencing variables of the ECDD process have been identified and set to fixed values. The applied voltage between the tool electrode and auxiliary anode is 100 V, the duty cycle is 50%, and the frequency is 1800 Hz. The KMnO₄ solution was freshly prepared before each experimental run to minimize any decomposition or precipitation. Visual inspection was carried out to ensure the absence of residue or sedimentation, and the electrolyte was continuously stirred during machining to maintain uniform dispersion of KMnO₄. In addition, the color intensity of the solution—serving as an indicator of oxidative strength—was periodically checked throughout the experiments. These measures ensured both the uniformity and stability of the oxidizing additive within the alkaline electrolyte during the machining trials. The variable under investigation is the combination of electrolytes, as listed in Table 1. Tool wear of brass, steel, and tungsten carbide was observed at different voltages, influenced by their varying melting points and compositions. The results showed that tungsten carbide exhibited lower wear at high voltages^42^. Therefore, this research utilized tungsten carbide as the tool electrode material, with dimensions of Ø1.75 × 75 mm. In general, the molarity (M) is calculated using the relationship among wt %, density of solution and molecular weight of solute (g/mol) as given in Eq. 1.

1 $$Molarity{\text{ }}\left( M \right) = \frac{{Weight{\text{ }}percentage{\text{ }}of{\text{ }}solute{\text{ }} \times {\text{ }}Density{\text{ }}of{\text{ }}solution{\text{ }} \times {\text{ }}10}}{{Molecular{\text{ }}weight{\text{ }}of{\text{ }}solute}}$$

**Table 1:** Various molarities of electrolytes used in ECDD.

Conditions (wt %)	Plain NaOH electrolyte (100%)	95% NaOH mixed with 5% KMnO_4_	50% NaOH + 50% KOH	47.5% NaOH + 47.5% KOH + 5% KMnO_4_
Molarity (M)	7.50	7.22	5.36	5.13

**Fig. 2:**
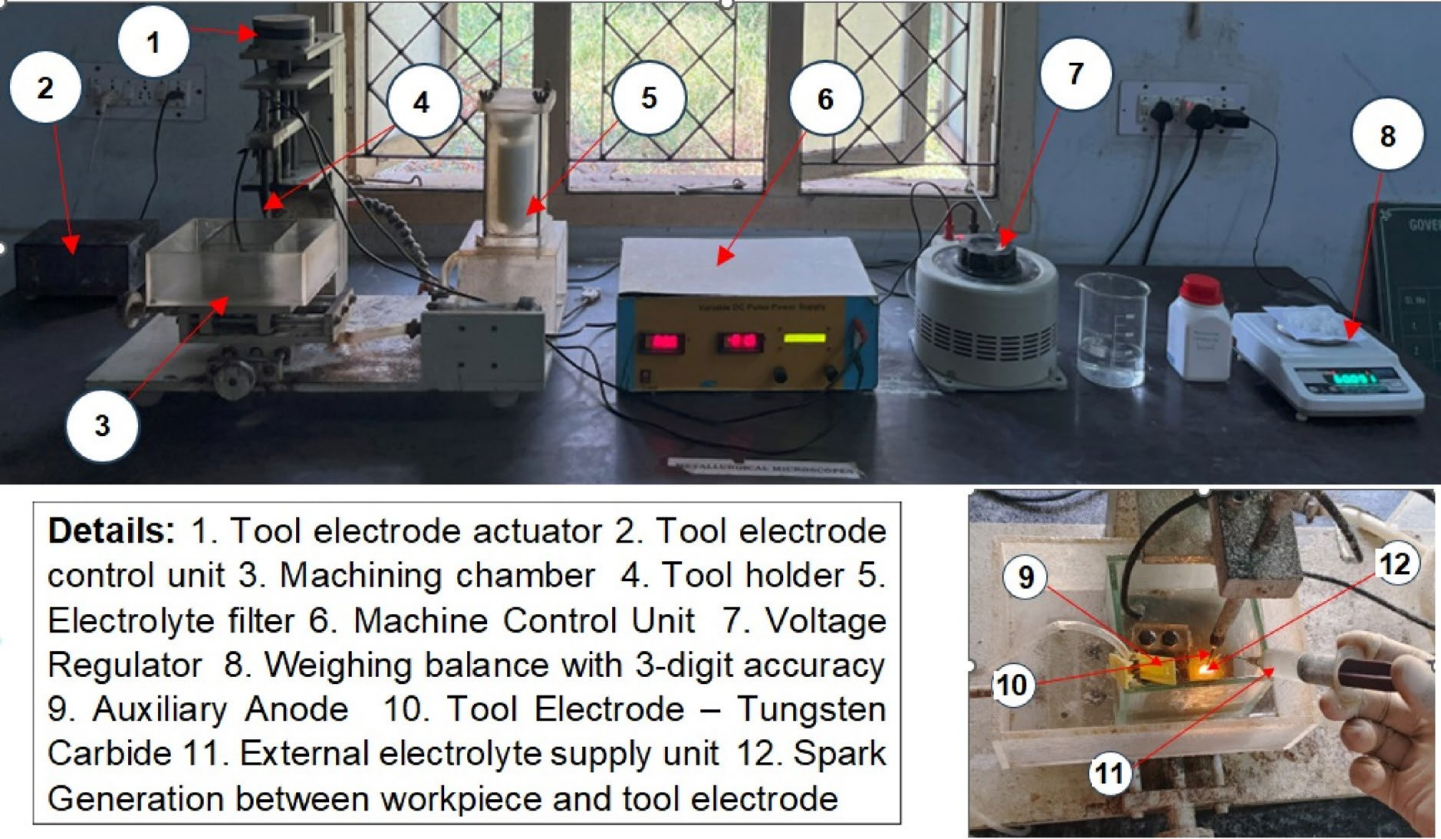
Indigenously developed electrochemical discharge drilling setup.

Figure 2 illustrates the schematic view of the indigenously developed ECDD setup. A DC power source with a pulse generator supplies voltage across the tool electrode (cathode) and the auxiliary electrode (anode). This potential difference induces electrolysis, forming bubbles near both electrodes. The workpiece is partially immersed in the electrolyte, and as the critical voltage is reached, these bubbles increase in number, forming a dense and coalescent gas film^43,44^.

The gas film generated between the tool and workpiece acts as a dielectric medium, preventing spark formation due to its insulating properties^45^. If the applied voltage surpasses the critical voltage, which is necessary to break the gas layer and initiate a spark, thermal erosion on the workpiece begins. In ECDD, the gas film operates in two regimes: discharge and hydrodynamic. Metal removal is most effective in the discharge regime, up to a depth of 300 μm. Beyond this depth, in the hydrodynamic regime, the gas film becomes unstable, resulting in reduced metal removal efficiency^46^. Axial tool wear is influenced by voltage, electrode diameter, electrolyte density, melting point of the tool material, spark intensity and configuration of the tool electrode design^47,48^. A key advantage of ECDD over ECM is its non-short circuit nature. In each experiment, the material loss of both the tool electrode and the workpiece was measured before and after machining using an electronic weighing machine with 0.001 g accuracy, allowing for the calculation of MRR and TWR^49^. During spark discharge, the tool electrode bombards the workpiece surface with electrons, raising its temperature and causing the workpiece to melt, facilitating metal removal. In ECDD of M.S sheet, the choice of electrolyte significantly influences the chemical reactions and overall machining process. Initially, Fe(OH)_2_ forms, which can further oxidize to Fe(OH)_3_. These hydroxides are typically insoluble and can affect machining by forming a passivating layer on the iron surface, requiring periodic removal. This has been done by using filter while circulating the electrolytes thorough the designed circuit. The KMnO_4_, a strong oxidizing agent, enhances the machining process by preventing passivating layer formation. The production of MnO_2_ helps break down the iron surface more effectively, improving machining efficiency. An aqueous solution of NaOH and KMnO_4_, characterized by high alkalinity, strong oxidizing nature, and high conductivity, effectively oxidizes iron surfaces, preventing passivation and aiding continuous material removal in ECDD processes.

## Results and discussion

Experiments results detailed in Table 2 for understanding the impacts of KMnO_4_ in ECDD on M.S sheets, focusing on hole dimensional accuracy in terms of over/undercut (%), MRR, and TWR. Tool wear, measured by tool electrode length reduction percentage, was also analyzed. The complex material removal process in ECDD has been theorized by various researchers.

**Table 2:** Impacts of KMnO_4_ in ECDD under various electrolytes conditions.

Sl. No	Conditions	Obtained hole diameter (mm)	Overcut/ Undercut (%)	MRR (mg/min)	TWR (mg/min)	Reduction in Length of Tool (%)
1.	Plain NaOH electrolyte	1.859	6.23	3.20	9.35	4.23
2.	95% NaOH mixed with 5% KMnO_4_	1.737	0.74	3.33	5.50	4.03
3.	50% NaOH + 50% KOH	2.712	54.97	1.43	6.25	9.33
4.	47.5% NaOH + 47.5% KOH + 5% KMnO_4_	2.595	48.29	1.72	6.00	6.66

**Fig. 3:**
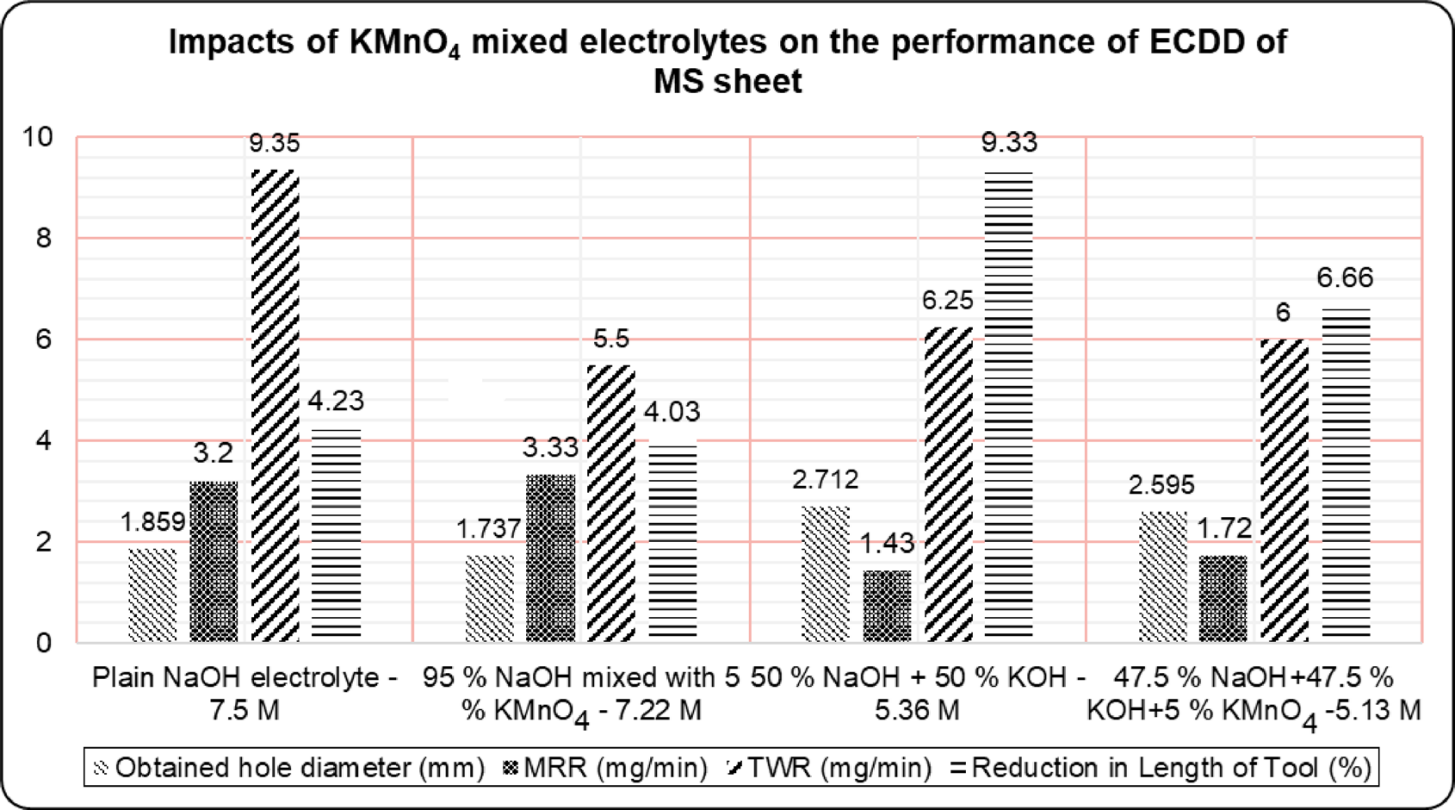
Impacts of KMnO4 mixed electrolytes on the performance of drilling in the ECDD of MS sheet.

One study suggests metal removal occurs through electrical sparks generated during the switching phenomenon rather than by breaking down the insulating gas layer^50^. Conversely, another researcher posits a mechanism involving local heating and chemical etching^51^. Additionally, the arc discharge valve theory proposes a mechanism for metal removal from the workpiece^52^. From this investigation, it appears that metal removal primarily results from localized workpiece melting due to heat penetration from sparks, involving gas film discharge, spark and chemical erosion, and atom-by-atom removal. The stability of the gas film, particularly during discharge and hydrodynamic regimes, significantly enhances ECDD performance, notably with the use of a 5% KMnO_4_ mixed NaOH electrolyte. According to Fig. 3, the study evaluated the hole diameter i.e. % of Overcut/undercut, MRR, TWR, and tool length reduction under different electrolyte combinations: plain NaOH (7.5 M), 5% KMnO_4_ mixed with NaOH (7.22 M), 50% NaOH and 50% KOH (5.36 M), and 5% KMnO_4_ mixed with 47.5% NaOH and 47.5% KOH. Each data point in Fig. 3 corresponds to the average of three independent experimental trials. The associated error was carefully evaluated and found to be within ± 5%.

### Effects of KMnO_4_ on dimensional accuracy of obtained drilled hole

This study aimed to achieve high-dimensional accuracy of hole in the ECDD process. This task is particularly challenging due to issues such as stray current erosion resulting in entry hole having a larger diameter than the inner part. Although the tool is insulated, it degrades due to high-intensity sparks generated during the ECDD process. Factors like bubble generation, gas film characteristics, the potential difference between the tool and auxiliary electrodes, and electrolyte conductivity significantly influence the quality of the holes produced in ECDD. To enhance accuracy, KMnO_4_, an oxidizing agent, was mixed with alkaline electrolytes (NaOH and KOH). To achieve precise holes, various electrolyte combinations were tested: plain NaOH, 95% NaOH with 5% KMnO_4_, 50% NaOH and 50% KOH, and 47.5% NaOH and KOH each with 5% KMnO_4_. Based on the results of the preliminary experiments, a maximum of 5% (wt.) KMnO_4_ was chosen for the above combinations of electrolytes.

Basically, KMnO₄ is a potent oxidizing agent that can facilitate the breakdown of passivating layers that form on the workpiece surface, such as oxides or other insulating films. In the ECDD process, the presence of KMnO₄ in the electrolyte accelerates the oxidation of these layers, preventing them from passivating the surface. This maintains the electrical conductivity of the machining gap and ensures a consistent MRR. When KMnO₄ is mixed with other electrolytes like NaOH/KOH, it undergoes reduction at the cathode while simultaneously causing the oxidation of metallic ions at the anode. The reactions between KMnO₄ and these bases create a more aggressive electrolytic environment. The reduction of MnO₄⁻ to MnO₂ in alkaline conditions results in the release of oxygen gas, which helps in maintaining a stable gas film around the tool electrode, enhancing the electrochemical discharge stability.

**Fig. 4:**
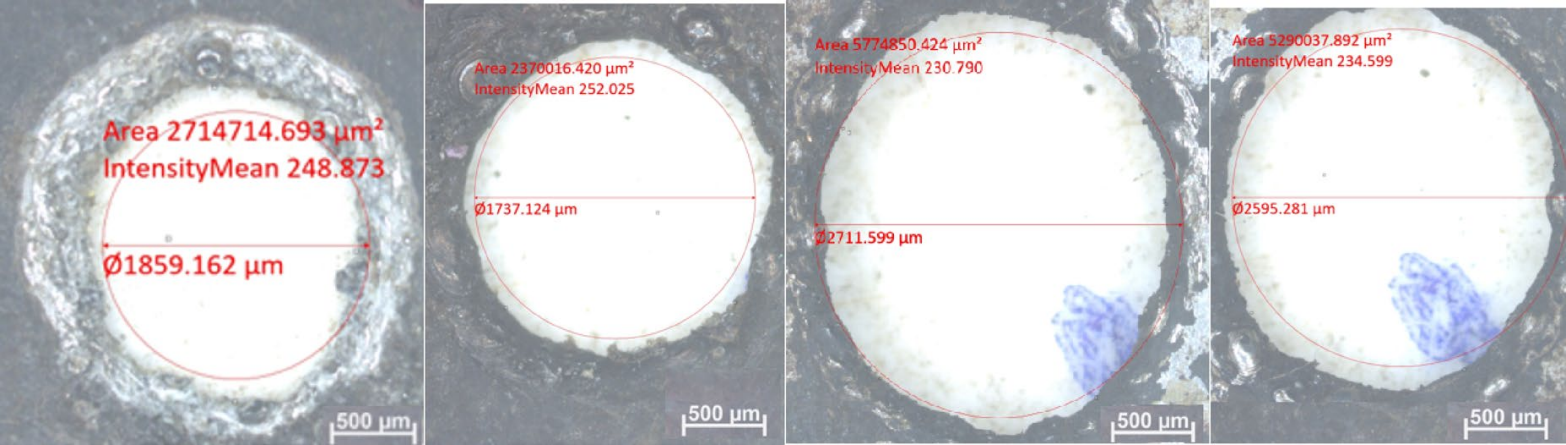
Optical microscope images of obtained holes using different electrolytes. (**a**) Plain NaOH electrolyte (**b**) 95% NaOH mixed with 5% KMnO (**c**) 50% NaOH + 50% KOH (**d**) 47.5%NaOH+ 47.5% KOH + 5% KMnO_4_

The mixed electrolyte’s ability to maintain a consistent current density across the machining gap helps in achieving better control over the electrochemical reactions. This prevents the formation of excessive heat and localized damage, minimizing the HAZs and enhancing the dimensional accuracy of the drilled holes. The novel electrolyte combination is specifically designed to optimize the balance between oxidative and reductive reactions, ensuring improved material removal, reduced passivation, and consistent drilling performance. Moreover, KMnO_4_ helps to enhance the thermal and electrical stability of the electrolytes, providing a stable drilling environment by preventing fluctuations in electrolyte properties. Figure 4 presents the optical images of the holes obtained using different electrolytes.

The recorded hole sizes were: 106.23% of the tool diameter with plain NaOH, 99.26% with 5% KMnO_4_ and 95% NaOH, 154.97% with NaOH and KOH, and 148.29% with 5% KMnO_4_, 47.5% NaOH, and 47.5% KOH against the tool electrode diameter of 1.75 mm. The use of KMnO_4_ mixed with NaOH resulted in a hole diameter of 1.737 mm, deviating by only 0.74% from the 1.75 mm tool electrode diameter. Additionally, combining NaOH and KOH with KMnO_4_ yielded the hole diameter of 2.595 mm, compared to 2.712 mm with plain NaOH and KOH, showing a 4.5% higher overcut. The combination of KMnO_4_ with NaOH also controlled stray corrosion by forming a thin layer over the tool surface during ECDD and prevented localized heating, resulting in dimensional accuracy. It is concluded that the addition of KMnO_4_ significantly improves the accuracy of holes in ECDD, meeting industrial demands for precise drilling in advanced engineering materials with high hardness, brittleness, and poor conductivity.

### Effects of KMnO_4_ on MRR and TWR

The introduction of KMnO₄, a strong oxidizing agent, modifies the local electrochemical environment by increasing the oxygen content in the machining gap, which facilitates the dissolution of metal ions from the workpiece surface. The presence of KMnO₄ in an alkaline medium, such as NaOH or KOH, prevents the formation of passivating oxide layers that can impede the metal removal process, thereby enhancing the overall material removal mechanism. In an alkaline solution like NaOH, KMnO₄ can undergo redox reactions that help stabilize the electrolyte, which in turn contributes to a more consistent and controlled drilling process. This stability reduces fluctuations in current density, improving the uniformity of the electrochemical discharges and enhancing the precision and quality of the machined holes. The reaction of KMnO_4_ in an alkaline medium can be represented as presented in Eq. 2^53^. Here, KMnO_4_ gets reduced to Na_2_MnO_4_, and this helps in maintaining the oxidative environment necessary for the ECDD process.

2 $$\:2KMn{O}_{4}+2{H}_{2}O+3NaOH\underrightarrow{}2KOH+2{Na}_{2}Mn{O}_{4}+2{H}_{2}{O}_{2}$$

The high pH of NaOH ensures the generation of hydroxide ions (OH⁻), which are essential for maintaining conductivity and facilitating chemical reactions during the drilling process. MRR generally depends on the applied voltage between the tool electrode and the auxiliary electrode, the thermal and electrical conductivity of the electrolytes, the stability of electrolytes during spark discharge, the pH value, and the formation of hydrogen and oxygen gas films due to the use of KMnO_4_. Among the four electrolyte combinations tested, results revealed that KMnO_4_ significantly improves the MRR. A higher MRR of 3.33 mg/min was achieved using 95% NaOH with 5% KMnO_4_, which is 4.1% higher than plain NaOH, 132.87% higher than the 50% NaOH + 50% KOH combination, and 93.6% higher than using 47.5% NaOH + 47.5% KOH + 5% KMnO_4_ electrolytes. Despite the higher pH value of plain NaOH, the stability of the electrolyte was better maintained when using KMnO_4_, resulting in a higher MRR than any other electrolyte combinations.

In this study, the improved tool life observed with KMnO₄ addition is attributed to its strong oxidizing nature, which is likely to facilitate the formation of a thin protective layer on the tool electrode surface. While direct experimental confirmation of this layer is not available in the present work, the performance trends summarized in Table 2 support this probable mechanism. The texture of the electrode surface plays a crucial role in bubble generation and coalescence, which are key factors in ensuring better MRR and TWR in ECDD. Generally, stray corrosion, instability in electrolyte conductivity, and spark intensity directly affect tool life. Therefore, the dimensional accuracy of the hole depends on the dimensional stability of the tool electrode during the ECDD process. The lowest TWR obtained was 5.5 mg/min using the 95% NaOH + 5% KMnO_4_ electrolyte, which is 70% lower compared to plain NaOH. This provides concrete evidence of KMnO_4_’s impact on achieving minimal TWR, as represented in Fig. 3.

The combination of NaOH and KOH did not significantly achieve the chosen lower TWR. Plain NaOH led to higher tool wear and notable tool electrode erosion despite NaOH and KOH being severe alkaline electrolytes with good ionic conductivities. Consequently, better MRR can be obtained by sacrificing tool electrode life. It is concluded that tool electrode life is reasonably improved when using KMnO_4_ mixed with NaOH electrolytes due to the formation of a protective thin layer on the tool electrode surface, making it suitable for ECDD applications. The percentage of reduction in tool length is also presented in Fig. 3. The experimental results revealed that the highest reduction in tool length occurred when using the 50% NaOH + 50% KOH electrolyte, followed by plain NaOH. The lowest reduction in tool length (4.03%) was observed when using the 95% NaOH + 5% KMnO_4_ electrolyte for making holes in ECDD, which is 132% lower.

### Effects of KMnO_4_ on HAZ

**Fig. 5:**
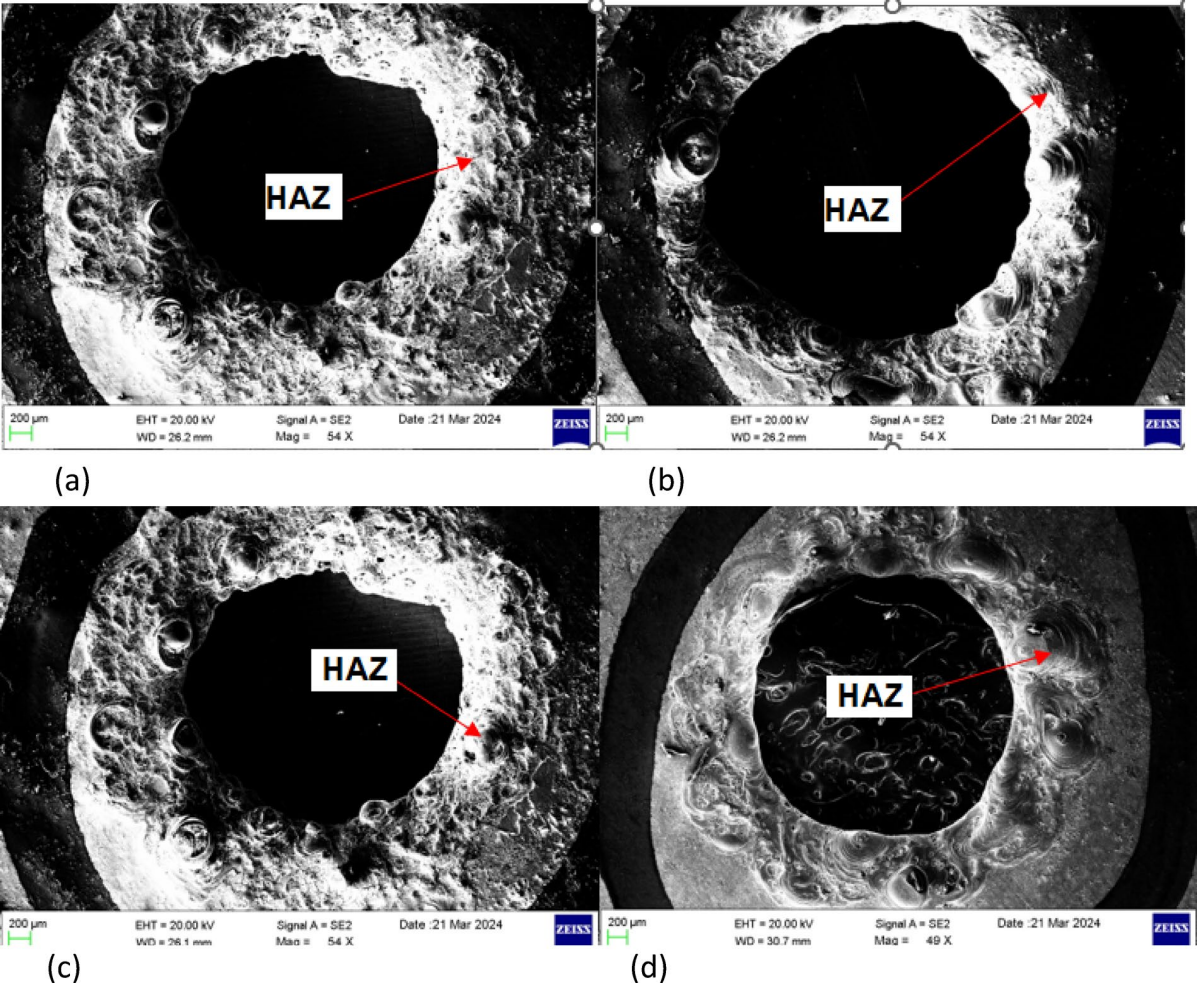
SEM images of holes with the heat affected zones while using different electrolytes with/without KMnO_4_. (**a**) Plain NaOH electrolyte (**b**) 95 % NaOH mixed with 5 % KMnO4 (**c**) 50 % NaOH + 50 % KOH (**d**) 47.5 % NaOH+47.5 % KOH+5 % KMnO4

The HAZ was identified as the peripheral ring-like region surrounding the hole, where microstructural changes due to thermal exposure were evident as shown in Fig. 5. The HAZ analysis was carried out using Scanning electron microscope (SEM) images for different electrolyte conditions: (a) Plain NaOH (b) 95% NaOH + 5% KMnO₄ (c) 50% NaOH + 50% KOH (d) 47.5% NaOH + 47.5% KOH + 5% KMnO₄. The scale bar (200 μm) indicates that the average HAZ width is in the range of ~ 100–250 μm around the periphery. From the qualitative SEM analysis, the results have been identified:

- Plain NaOH (a): A wide and irregular HAZ was observed, indicating higher thermal damage and unstable discharge conditions.
- 95% NaOH + 5% KMnO₄ (b): The narrowest and most uniform HAZ was observed in this condition, suggesting the beneficial role of KMnO₄ in stabilizing discharge and reducing excess heating.
- 50% NaOH + 50% KOH (c): The HAZ appeared moderately reduced compared to plain NaOH, reflecting improved ionic conductivity and controlled discharge spread.
- 47.5% NaOH + 47.5% KOH + 5% KMnO₄ (d): A comparatively narrower HAZ was seen in this condition, highlighting the effect of mixed electrolytes with KMnO₄ in minimizing thermal damage.

Generally, the use of alkaline electrolytes such as NaOH and KOH enhances MRR but sacrifices material properties at the entrance area of the electrochemically drilled workpieces. This effect is also seen with the combination of NaOH and KOH electrolytes, as evidenced by the SEM images in Fig. 5c. The HAZ is greater when using plain NaOH and combined NaOH and KOH electrolytes^54^. However, the use of a KMnO_4_ mixed NaOH electrolyte solution results in better hole size and MRR with minimal HAZ.

The KMnO_4_ reacts with alkene bonds, shifting electrons and creating bonds to form a cyclic manganese compound. In the next stage, NaOH enters the reaction and hydrolyzes the bonds to form a diol under cold conditions. However, during continuous spark generation in the machining gap, this reaction leads instead to the formation of a carbonyl group. In ECDD, the interaction between KMnO₄ and NaOH electrolytes is crucial, particularly for the HAZ. When KMnO₄ is mixed with NaOH, it acts as a strong oxidizing agent, forming a more stable and conductive electrolyte. This stability ensures consistent electrical discharges, leading to more precise material removal. The oxidizing properties of KMnO₄ may also contribute to the formation of a protective layer on the tool electrode, which in turn can reduce tool wear. Additionally, KMnO₄ presence improves breakdown voltage, enhancing the sparking mechanism. This controlled sparking reduces thermal damage to the workpiece, thereby minimizing the HAZ. Consequently, localized heating is confined to a smaller area, improving the dimensional accuracy of the drilled features and reducing unwanted thermal effects like micro-cracking and surface oxidation. Overall, using KMnO₄ mixed with NaOH electrolyte improves the ECDD process in terms of MRR and TWR, significantly reducing adverse impacts on the HAZ and leading to better dimensional accuracy with minimal HAZ. The presence of carbonyl groups enhances heat dissipation from the tool surface.

It is well established that KMnO₄ oxidation can introduce carbonyl and other oxygenated functional groups on surfaces, which in turn improve electron mobility and thermal conductivity^55^. In the present experiments, the observed reduction in tool wear is attributed to the probable formation of carbonyl groups on the tool electrode surface during the ECDD process. This arises from the oxidative interaction of KMnO₄ with organic residues or hydrocarbon species generated under spark discharge conditions. Under continuous sparking in an alkaline environment, KMnO₄ tends to oxidize unsaturated bonds, leading to the formation of carbonyl functionalities such as aldehydes and ketones on the electrode surface or at the electrolyte–electrode interface. These polar groups are likely to enhance thermal conductivity and promote more efficient heat dissipation from the tool surface, thereby reducing localized overheating and minimizing tool wear.

Such surface chemical modifications have been demonstrated to increase surface energy and thermal stability, as reported in studies on electrochemical surface oxidation and modification. Carbonyl groups improve electron mobility and contribute to more uniform and stable sparking by stabilizing the electrolyte-electrode interface, which in turn reduces erratic tool wear caused by inconsistent discharge intensities^56^. Efficient heat dissipation reduces thermal stress and prevents the tool from overheating, which is one of the key factors in minimizing tool wear during the ECDD process. Carbonyl groups can also influence the sparking process by creating a more uniform and stable discharge environment. This uniformity minimizes the intensity and irregularity of electrical sparks, thereby reducing erratic tool wear caused by inconsistent sparking. Consequently, the use of KMnO_4_ with alkaline electrolytes, specifically NaOH, improves the lifespan and geometrical stability of the tool, enhancing the overall performance of the ECDD process.

### Potential limitations of using KMnO₄ with 95% NaOH in industrial applications

While the addition of 5% KMnO₄ to 95% NaOH electrolyte offers significant improvements in ECDD performance, certain limitations and challenges must be considered for broader industrial adoption. Firstly, the chemical stability of KMnO₄ in highly alkaline solutions can be compromised over extended use due to its strong oxidizing nature, potentially leading to gradual depletion of active species and requiring frequent electrolyte replacement or replenishment to maintain machining consistency.

Secondly, electrode contamination is a concern as KMnO₄ and its reduction products, such as manganese dioxide (MnO₂) can deposit on the tool electrode surface or workpiece, potentially affecting electrical conductivity and the uniformity of spark discharges. This buildup may necessitate periodic cleaning or additional filtration systems in the electrolyte circulation loop.

Furthermore, the intensity of electrical discharges can be influenced by the electrolyte composition; strong oxidizers like KMnO₄ would intensify sparking, if not properly controlled, can lead to uneven wear, micro-cracks, or unwanted surface alterations, thus requiring precise parameter optimization and process monitoring.

Safety and environmental considerations must also be addressed. KMnO₄ is a hazardous oxidizing chemical, presenting risks such as chemical burns, toxicity, and environmental harm if mishandled or improperly disposed of. Industrial operations must implement strict safety protocols and ensure responsible waste management to mitigate these risks. Additionally, handling highly concentrated NaOH demands caution due to its corrosive nature.

Overall, while KMnO₄ mixed with 95% NaOH demonstrates clear process benefits, these limitations highlight the need for further research into electrolyte stability, system design for contamination control, spark intensity regulation, and adherence to safety and environmental standards to enable safe, sustainable, and efficient industrial usage.

## Conclusions

The electrochemical discharge drilling (ECDD) process is a hybrid technique essential for creating precise holes in advanced engineering materials such as metal sheets, composites, and ceramics. This study aimed to improve ECDD’s efficiency by introducing potassium permanganate (KMnO_4_) mixed with sodium hydroxide (NaOH) and potassium hydroxide (KOH) electrolytes. The primary focus was on enhancing the material removal rate (MRR), reducing the tool wear rate (TWR), and achieving better dimensional accuracy while minimizing the heat-affected zone (HAZ). The major key findings are listed below:

- The introduction of 5% KMnO_4_ in a 95% NaOH electrolyte resulted in a hole diameter deviation of only 0.74% from the 1.75 mm tool electrode, significantly improving accuracy.
- KMnO_4_ minimized stray corrosion and localized heating, leading to enhanced dimensional precision compared to plain NaOH and combined NaOH-KOH electrolytes.
- The highest MRR of 3.33 mg/min was achieved with 95% NaOH with 5% KMnO_4_, representing a 4.1% increase over plain NaOH and significantly higher than other combinations.
- KMnO_4_ stabilized the electrolyte, maintaining its stability during the spark discharge process, thereby enhancing MRR.
- The lowest TWR of 5.5 mg/min was observed with 95% NaOH with 5% KMnO_4_, a 70% reduction compared to plain NaOH.
- The reduced tool wear observed in this study might be attributed to probable tool surface modifications, including the formation of protective oxide layers and oxygenated functional groups. Such modifications can enhance heat dissipation and stabilize the tool–electrolyte interface, thereby contributing to improved tool performance.
- The interaction between KMnO_4_ and NaOH improved electrolyte stability, resulting in lower HAZ.
- The demonstrated enhancement of ECDD performance using KMnO₄ mixed with 95% NaOH on mild steel suggests promising potential for adaptation to other engineering materials such as stainless steel, titanium alloys, and nickel-based superalloys. These materials are widely used in aerospace, biomedical, and automotive industries, where precise micro-hole machining is critical. The strong oxidizing nature of KMnO₄ could similarly prevent passivation layer formation on these materials’ surfaces, potentially improving MRR and dimensional accuracy.

## Data Availability

The datasets used and/or analyzed during the current study are available from the corresponding author upon reasonable request.

## Abbreviations

ECDD: Electrochemical discharge drilling
ECDM: Electrochemical discharge machining
ECD: Electrochemical drilling
EDD: Electrical discharge drilling
UA: ECDM-Ultrasonic assisted Electrochemical discharge machining
MRR: Material removal rate
TWR: Tool wear rate
HAZ: Heat affected zone
MEMS: Micro-electro-mechanical systems
NaOH: Sodium hydroxide
KOH: Potassium hydroxide
KMnO_4_: Potassium permanganate
NaCl: Sodium chloride
NaNO_3_: Sodium nitrate
MnO_2_: Manganese di-oxide
SEM: Scanning electron microscope
Al_2_O_3_: Aluminum Oxide
RM: WECSM-Reversible Motion-Wire Electrochemical Spark Machining
Fe(OH)_2_: Ferrous Hydroxide
M: Molarity
